# Supplementary material for: A Critical View of the Application of the APEX Software (Aqueous Photochemistry of Environmentally-Occurring Xenobiotics) to Predict Photoreaction Kinetics in Surface Freshwaters
Source: Molecules. 2019 Dec 18;25(1):9. doi: 10.3390/molecules25010009 (PMC7017383; doi:10.3390/molecules25010009)
Supplement: Supplementary file 1 [file molecules-25-00009-s001.zip › 3.2.4_gcc-4.4.0.zip!/3.2.4_gcc-4.4.0/3.2.4_gcc-4.4.0/doc/octave/html/interpreter/Service.html]

Service - Untitled


Previous: Sending Patches,
Up: Trouble


---

### E.7 How To Get Help with Octave

The mailing list help@octave.org exists for the discussion of
matters related to using and installing Octave. If would like to join
the discussion, please send a short note to
help**-request**@octave.org.

**Please do not** send requests to be added or removed from the
mailing list, or other administrative trivia to the list itself.

If you think you have found a bug in the installation procedure,
however, you should send a complete bug report for the problem to
bug@octave.org. See Bug Reporting, for
information that will help you to submit a useful report.
